# Marine-Derived Compounds for CDK5 Inhibition in Cancer: Integrating Multi-Stage Virtual Screening, MM/GBSA Analysis and Molecular Dynamics Investigations

**DOI:** 10.3390/metabo13101090

**Published:** 2023-10-18

**Authors:** Tagyedeen H. Shoaib, Mohammed A. Almogaddam, Yusra Saleh Andijani, Samaher Ahmad Saib, Najwa Mahmoud Almaghrabi, Abdulaziz Fahad Elyas, Rahmah Yasin Azzouni, Ehda Ahmad Awad, Shaimaa G. A. Mohamed, Gamal A. Mohamed, Sabrin R. M. Ibrahim, Hazem G. A. Hussein, Wadah Osman, Ahmed Ashour, Asmaa E. Sherif, Abdulrahim A. Alzain

**Affiliations:** 1 Department of Pharmaceutical Chemistry, Faculty of Pharmacy, University of Gezira, Wad Madani 21111, Sudan; shoaibth37@hotmail.com (T.H.S.); almogaddamma@gmail.com (M.A.A.); 2 Department of Pharmacology and Toxicology, College of Pharmacy, Taibah University, Al-Madinah Al-Munawwarah 30078, Saudi Arabia; yandijani@taibahu.edu.sa; 3 College of Pharmacy, Taibah University, Medina 42353, Saudi Arabia; samahersaib@hotmail.com; 4 Pharmaceutical Care, King Abdullah Medical Complex, MOH, Jeddah 23816, Saudi Arabia; najwamghrbi@gmail.com; 5 Emergency Medical Services Department, Madinah National Hospital, Madinah 11461, Saudi Arabia; abdulazizelyas9@gmail.com; 6 King Faisal Specialist Hospital & Research Center, Al-Madinah Al-Munawwarah 42523, Saudi Arabia; rahmaAzzony@gmail.com; 7 Prince Mohammed Bin Abdulaziz Hospital-Al Madinah Al Munawarah-NGHA, Ministry of National Guard Health Affairs, Kingdom of Saudi Arabia, Riyadh 41511, Saudi Arabia; Ehdaa.ahmad.awadh@gmail.com; 8 Faculty of Dentistry, British University, El Sherouk City, Suez Desert Road, Cairo 11837, Egypt; shaimaag1973@gmail.com; 9 Department of Natural Products and Alternative Medicine, Faculty of Pharmacy, King Abdulaziz University, Jeddah 21589, Saudi Arabia; gahussein@kau.edu.sa; 10 Preparatory Year Program, Department of Chemistry, Batterjee Medical College, Jeddah 21442, Saudi Arabia; 11 Department of Pharmacognosy, Faculty of Pharmacy, Assiut University, Assiut 71526, Egypt; 12 Preparatory Year Program, Batterjee Medical College, Jeddah 21442, Saudi Arabia; hazemgamal2005@gmail.com; 13 Department of Pharmacognosy, Faculty of Pharmacy, Prince Sattam Bin Abdulaziz University, Al-kharj 11942, Saudi Arabia; w.osman@psau.edu.sa (W.O.); ahmed.mohamed@psau.edu.sa (A.A.); ae.sherif@psau.edu.sa (A.E.S.); 14 Department of Pharmacognosy, Faculty of Pharmacy, University of Khartoum, Al-Qasr Ave., Khartoum 11111, Sudan; 15 Department of Pharmacognosy, Faculty of Pharmacy, Mansoura University, Mansoura 35516, Egypt

**Keywords:** CDK5, cancer, marine compounds, drug discovery, molecular docking, molecular dynamics, life below water, health and wellbeing

## Abstract

Cyclin-dependent kinase 5 (CDK5) plays a crucial role in various biological processes, including immune response, insulin secretion regulation, apoptosis, DNA (deoxyribonucleic acid) damage response, epithelial−mesenchymal transition (EMT), cell migration and invasion, angiogenesis, and myogenesis. Overactivation of CDK5 is associated with the initiation and progression of cancer. Inhibiting CDK5 has shown potential in suppressing cancer development. Despite advancements in CDK5-targeted inhibitor research, the range of compounds available for clinical and preclinical trials remains limited. The marine environment has emerged as a prolific source of diverse natural products with noteworthy biological activities, including anti-cancer properties. In this study, we screened a library of 47,450 marine natural compounds from the comprehensive marine natural product database (CMNPD) to assess their binding affinity with CDK5. Marine compounds demonstrating superior binding affinity compared to a reference compound were identified through high-throughput virtual screening, standard precision and extra-precision Glide docking modes. Refinement of the selected molecules involved evaluating molecular mechanics–generalized born surface area (MM/GBSA) free binding energy. The three most promising compounds, (excoecariphenol B, excoecariphenol A, and zyzzyanone B), along with the reference, exhibiting favorable binding characteristics were chosen for molecular dynamics (MD) simulations for 200 nanoseconds. These compounds demonstrated interaction stability with the target during MD simulations. The marine compounds identified in this study hold potential as effective CDK5 inhibitors and warrant subsequent experimental validation.

## 1. Introduction

Cancer is the world’s second most prevalent cause of death after coronary heart disease (CHD), and it is expected that it will become the first in the upcoming four decades [1]. Worldwide, the number of cancer-related deaths was 10 million out of 19.3 million according to updated WHO GLOBOCAN estimates. In descending order of fatality, lung, colorectal, liver, stomach, and breast cancers ranked as the most deadly forms of cancer [2].

CDK5 is a 292-amino-acids sequence protein that descends from the cyclin protein kinase (CDK) family alongside another 20 members [3,4]. For decades, CDKs have been projected as cell cycle regulators; they control G1 to S phase transition depending on their activation by cyclin [5]. CDK5 structure is 60% similar to other CDKs (i.e., CDK1 and CDK2 [3]); however, CDK5 is non-cyclin dependent since it lacks the amino acid motif that interacts with cyclin, and is not considered a direct cell cycle regulator [6,7]. CDK5 consists of an ATP (adenosine triphosphate) binding pocket intercalated between two lobes: the N-lobe which contains a 40 s subunit that is responsible for interaction with CDK5 activators, and the C-lobe that moderates ATP and substrate binding to the N-terminus [8,9]. 

Up-to-date research established an evident correlation between CDK5 aberrant activation and/or overexpression and tumorigenesis, mutagenesis dissemination, and cancer disease progression in a variety of cancers [10,11]. CDK5 has unignorable role in tumor progression and metastasis in numerous cancers, including head and neck squamous cell carcinomas, hepatocellular carcinoma, prostate cancer, medullary thyroid carcinoma, ovarian cancer, and chemotherapy-resistant cervical tumors of the uterine cervix and the bladder [12,13,14,15,16,17,18,19,20,21].

Despite the extensive studies that correlating CDK5 expression and cancer occurrence, drug therapies that selectively inhibit CDK5 were not available until very recently [22]. Selective targeting of CDK5 is extremely difficult due to the high degree of similarity with other CDKs [23]. Pan CDK inhibitors remain the chief therapeutic option for the treatment of CDK5-related illnesses [22]. First-generation pan CDK inhibitors, Roscovitine and flavopiridol, have entered Phase II clinical trials. However, they exhibited limited efficacy, and their side effects were deleterious. The second-generation pan CDKI, Dinaciclib, in phase II/III is far more potent than the first generation, but it displays low inhibitory activity against CDK5 and disastrous off-target toxicity [24,25]. At present, GFB-12811, modified thiazoles, and pyrido (3,2, *d)* pyridines have proven their selective CDK5 inhibitory properties. Nonetheless, they are still in the experimental phase [23,26,27].

Marine-based drug discovery has emerged as an exciting field in modern drug research and development since the discovery of the first marine derivative, MNP spongothymidine [28]. Currently, a lot of marine-derived medications have gained FDA approval (Ziconotide, Trabectedin, Eribulin, etc.), and many of them have entered clinical trials. Furthermore, a comprehensive marine natural product database (CMNPD) with more than 30,000 derivatives has become available for research purposes [29]. 

Computer-aided drug design is a multidisciplinary approach that intends to apply the fundamentals of computer sciences and software algorithms to assist the drug discovery process in terms of time and cost reduction [30,31,32]. 

The primary aim of this research endeavor is to exploit the various computer-aided drug design methodologies to systematically identify novel lead compounds demonstrating exclusive inhibitory activity against CDK5 within the context of comprehensive marine natural product database. Through comprehensive computational analyses and simulations, we seek to unravel promising candidate compounds with the potential to modulate CDK5, thus advancing our understanding of their therapeutic utility in CMNPD.

## 2. Materials and Methods

In this study, the Maestro version 12.8 software suite of Schrödinger and Desmond v6.5 module were simultaneously utilized to implement computational work to CDK5 protein structure analyses and the collected marine natural products. 

### 2.1. Protein Preparation

The 3D structure of the crystalized CDK5 enzyme (PDB ID: 7VDQ) bound to a known potent selective inhibitor was retrieved from the RCSB Protein Data Bank [33]. The structure was submitted to the protein preparation wizard tool in Maestro 12.8 software suite. Correspondingly, the protein structure was preprocessed. Throughout this step, hydrogen atoms were added, bond orders for the constitutional amino acid residues were assigned, heteroatoms and solvent molecules beyond 5 Ǻ were deleted, hydrogen bonds were optimized, and zero-order bonds with metals were generated. In addition, the function of prime module Maestro was recruited to add the omitted side chains and loops. Eventually, the energy of the preprocessed and optimized structure was minimized based on OPLS force field energy parameters [34,35].

### 2.2. Ligand Preparation

A set of 47,450 2D structures was obtained from a comprehensive marine natural product database. Upon that, the default settings of LigPrep tool in Maestro 12.8 software suite were employed to prepare the collected ligands and decrease probable computational errors [36]. Subsequently, precise, energy-minimized, and refined 3D chemical assemblies were obtained, probable mistakes in the structures of the ligands library were removed, and the possible ionization states were generated at the physiological pH (7_+/−2_). 

### 2.3. Molecular Docking

Following preparation of the collected dataset, the default settings of the Glide receptor grid generator tool in Maestro 12.8 were employed to create a cubic grid of 20 Ǻ, taking the centroid of the co-crystalized inhibitor of CDK5 as a reference place for ligand–receptor fitting [37].

The Glide module in Maestro 12.8 was implemented. The Glide module is a well-established docking platform that applies the Monte Carlo technique for ligand–receptor sampling and glide docking score (GScore), which is a modified form of ChemScore, as an energy scoring function retrieving the energy functions from the OPLS force field. The Glide module provides three modes of docking with different degrees of thoroughness and time labor [38]. High-throughput virtual screening (HTVS) represents the fastest mode; however, it uses a more forgiving scoring function and less extensive sampling techniques. It has been exploited to exclude compounds that possess poor receptor complementarity and reduce the number of tested molecules in the subsequent steps of the docking process. Thereafter, the top scoring molecules were subjected to Glide standard-precision docking (SP) followed by extra-precision (XP) docking that incorporates more extensive sampling and reliable scoring functions, and the top ten compounds were selected for further analysis based on their Glide docking score values [39,40,41]. 

### 2.4. MM/GBSA Binding Free Energy Calculation

The prime molecular mechanics–generalized born area (MM/GBSA) tool of Maestro is designed to calculate the binding free energy of the system based on the energy difference between the unbound minimized ligand–protein complex and the minimized complex after ligand binding, as depicted by the equation using OPLS_2005 as the force field and the VSGB2.0 solvent model [42].


∆G(bind) = E_complex_(minimized) − E_ligand_(minimized) + E_receptor_(minimized) (1)

The ten poses that have been preserved from the Glide XP docking output file underwent MM/GBSA calculations using the Prime module in Maestro version 12.8 software package and binding free energy (expressed as MM/GBSA ∆Gbind), and the energetic contribution in the ligand–receptor complex was estimated.

### 2.5. ADME and Toxicity Prediction 

Early ADMET (absorption, distribution, metabolism, excretion, and toxicity) pro-filing is of a paramount essence within the drug discovery journey. Among the clinical trial data between 2010 and 2017, poor drug-like properties aborted the discovery of approximately 10% to 15%, whereas undesirable toxicity prohibited the development of 30% of newly discovered lead molecules [43]. In this study, we exploited the QikProp tool in the Maestro 12.8 software suite of Schrödinger to forecast the pharmacokinetic profile of the refined molecules in MM/GBSA calculations. Afterward, Pro Tox II webserver was recruited to get a deeper look into the toxicological tendencies of the selected compounds. The selection of these tools was guided with their light computational requirements and tremendous simulation power [44,45].

### 2.6. Molecular Dynamics Simulation

Molecular dynamics (MD) simulation is one of the most influential trends in computational drug design. MD implicates molecular mechanics concepts, Newton’s law of motion, and mathematical principles to simulate particulate movement, and gives a thorough analysis of their interaction forces while considering the physiological barriers that face the drug molecule inside the human body. Moreover, MD investigates the binding energy, unbinding energy, and conformational changes underlying interactions between the molecules and their receptors [46].

From MM/GBSA binding free energy results, the three compounds with the lowest binding energy were subjected to MD simulation to assess the stability of their interaction with CDK5 (PDB ID: 7VDQ). The academic version of the Desmond version 6.5 software package of D.E Shaw research was used to run the simulation process. First, the physiological simulation system was created by placing the nominated ligands with CDK5 3D structure in an orthorhombic box with the dimensions of 10 × 10 × 10 Ǻ, and transferable intermolecular potential three-point TIP3P was added to the box as a solvent model. Furthermore, the ions of Na+ and Cl− were added to maintain the electrostatic neutralization of the system and attain the physiologic concentration of 150 mM. The system energy was minimized using the energy parameters of the OPL3Se force field. To generate a state of equilibrium, an isothermal–isochoric (NVT) ensemble was used to maintain at 300 K accompanied by an isothermal–isobaric (NPT) ensemble to attain fixed values for pressure and temperature. For the sake of maintaining conditions of steady pressure and temperature, the Nose–Hoover chain thermostat and the Martyna–Tobias–Klein barostat were adopted. Finally, molecular dynamics simulation was run for 200 ns and the resultant trajectories were analyzed via Maestro’s Simulation Interaction Diagram panel and evaluated according to their root mean square deviation RMSD and root mean square fluctuation RMSF [47].

## 3. Results and Discussion

### 3.1. Molecular Docking and MM/GBSA Analysis

Molecular docking stands as an efficient and economically viable method within the realm of computational drug design. It serves the purpose of identifying and evaluating essential molecular interactions occurring between ligands and receptors [48,49]. In the present work, a collection of 47,450 compounds extracted from marine sources [29] underwent molecular docking analysis against CDK5, employing a high-throughput virtual screening (HTVS) mode. Subsequently, the top 1000 compounds were subjected to a second round of docking using the standard-precision (SP) mode, and the top 100 compounds from this selection were further evaluated using the extra-precision (XP) mode. The XP mode is known to be the most accurate and precise among all other modes, despite being computationally extensive. Hence, for the primary filtration of compounds, we relied on their XP docking scores. The scoring function that is built in with Glide utilizes both empirical and force-field-based factors for calculating the binding energy and filtering the best pose of docking [41,50]. The co-crystallized reference molecule, which is already reported as a potent and selective CDK5 inhibitor [23], presented a very high docking score of −13.516 kcal/mol, as summarized in Table 1. Comparably, the highest-ranked inhibitors displayed a scoring spectrum, ranging from −12.015 kcal/mol to as low as −13.863 kcal/mol, showcasing a diversity of binding affinities across the compounds studied. 

**Figure 1:**
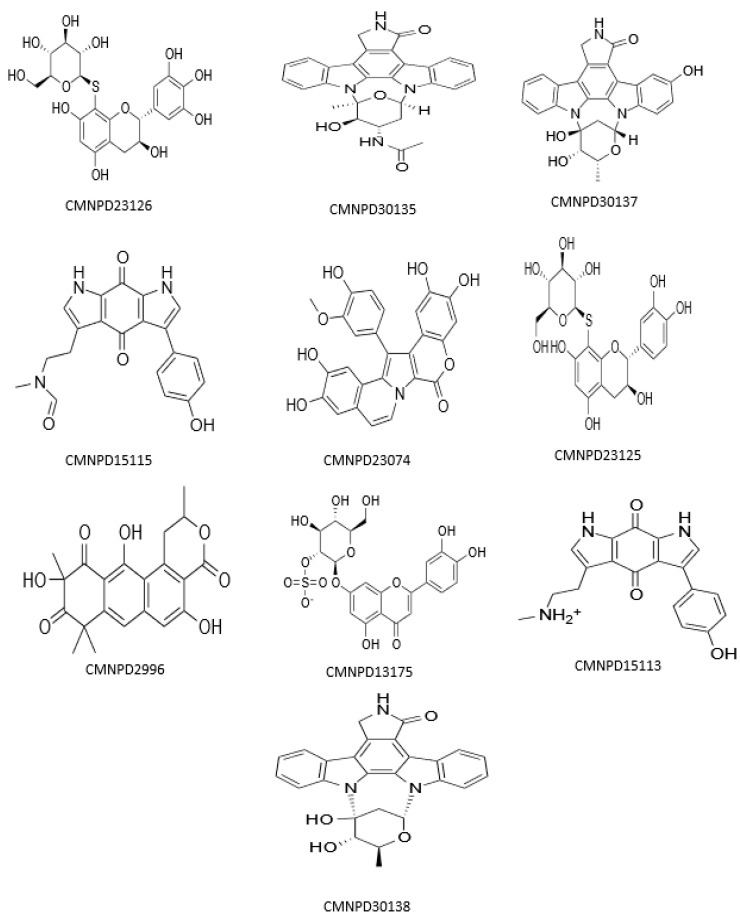
Chemical structures of the top 10 docked compounds with their CMNPD identification codes.

It can be seen from the docking scores that there are variable binding affinities conveyed by molecular docking. However, it is evident that further refinements are necessary for these compounds before proceeding with additional processing or analysis. One example of refinement means is the use of MM/GBSA (molecular mechanics/generalized born surface area). Numerous investigations have consistently demonstrated that MM/GBSA generally surpasses the scoring functions employed in docking algorithms when it comes to accurately ranking ligands based on their binding affinity [51,52,53]. To achieve the goal of safely fine-tuning the docked compounds, we used the Prime module to calculate the MM/GBSA binding free energies. As reported in Table 1, the co-crystallized reference inhibitor had a binding energy of −74.93 kcal/mol and the rest of the top 10 compounds presented values ranging from −47.20 kcal/mol to −83.78 kcal/mol. Figure 1 summarizes the chemical structures of the top 10 compounds. 

To proceed with more in-depth analysis, we chose the three most promising candidates: excoecariphenol B, excoecariphenol A, and zyzzyanone B. These compounds exhibit MM/GBSA values of −83.78 kcal/mol, −71.58 kcal/mol, and −71.05 kcal/mol, respectively.

**Figure 2:**
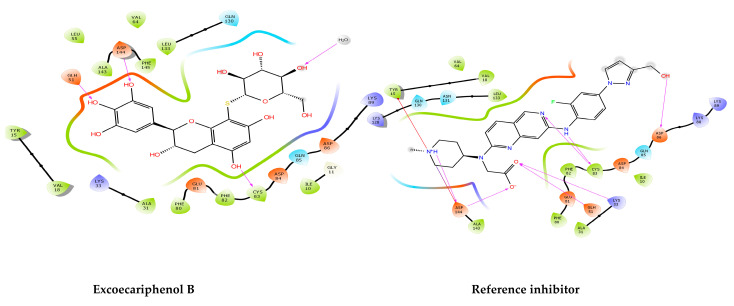

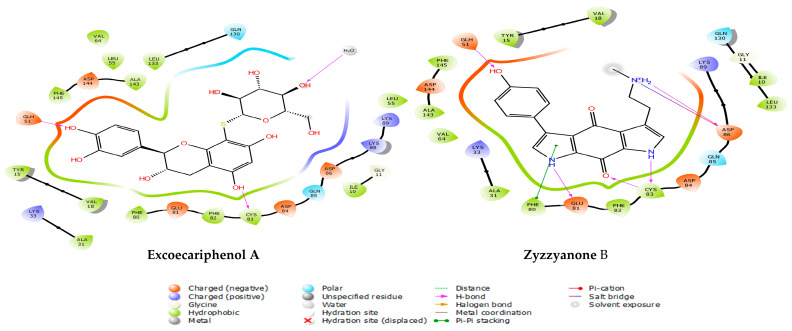
The 2D interactions of the top inhibitors and the co-crystallized reference inhibitor with the CDK5 enzyme (PDB ID: 7VDQ). The bottom legends identify the types of interaction and bonds with their corresponding color codes.

According to the summary of 2D interactions observed in Figure 2 and detailed in Table 2, it is evident that the co-crystallized inhibitor effectively replicated critical hydrogen bonding interactions with specific amino acid residues, including Lys33, Gln51, Cys83, Asp86, and Asp144. Additionally, it established a noteworthy salt bridge interaction with Asp144 and a Pi-cation interaction with Tyr15. 

**Table 1:** Docking scores of the top 10 ranking compounds with their MM/GBSA free binding energy results.

Compound ID	Name	Docking Score	XP G Score	MM/GBSA dG Bind	Taxonomy
**CMNPD23126**	excoecariphenol B	−13.343	−13.343	−83.78	*Excoecaria agallocha* [54]
**Co-crystallized reference**	2-[[7-[[2-fluoranyl-4-[3-(hydroxymethyl) pyrazol-1-yl] phenyl] amino]-1,6-naphthyridin-2-yl]-(1-methylpiperidin-4-yl) amino] ethanoic acid	−13.516	−13.516	−74.93	-
**CMNPD23125**	excoecariphenol A	−12.018	−12.018	−71.58	*Excoecaria agallocha* [54]
**CMNPD15113**	zyzzyanone B	−12.791	−12.791	−71.05	*Zyzzya fuliginosa* [55,56]
**CMNPD15115**	zyzzyanone D	−12.058	−12.058	−69.36	*Zyzzya fuliginosa* [55,56]
**CMNPD23074**	lamellarin A5	−12.015	−12.015	−61.95	*Didemnum* species [57]
**CMNPD30137**	streptocarbazole E	−13.863	−13.863	−60.50	*Streptomyces* species [58]
**CMNPD30135**	3′-O-demethyl-4′-N-demethyl-4′-N-acetyl-4′-epi-staurosporine	−12.547	−12.547	−56.15	*Streptomyces* species [59]
**CMNPD30138**	streptocarbazole C	−12.190	−12.190	−53.82	*Streptomyces* species [58]
**CMNPD13175**	thalassiolin A	−12.310	−12.310	−51.22	*Thalassia testudinum* [60]
**CMNPD2996**	2-hydroxygarvin B	−12.449	−12.449	−47.20	*Garveia annulata* [61]

Notably, excoecariphenol B demonstrated its potential by forming hydrogen bonds with Gln51, Cys83, and Asp144, contributing to its binding affinity. This compound is a flavan-3-ol that belongs to the structural superfamily of phenylpropanoids and polyketides, and it is known to be produced by the mangrove plant *Excoecaria agallocha*. The extracts of this plant (excoecariphenols A–D) have previously been reported to act against hepatitis C virus [54].

Conversely, the other flavan-3-ol, excoecariphenol A, exhibited hydrogen bonding interactions with Gln51 and Cys83, underscoring its capacity to engage with the receptor. Meanwhile, the third compound, zyzzyanone B, displayed a broader spectrum of interactions, including hydrogen bonds with Gln51, Glu81, Asp86, and Cys83. It also featured a salt bridge interaction with Asp86 and a Pi–Pi stacking interaction with Phe80, further emphasizing its binding versatility. Zyzzyanone B is a pyrroloindole that descends from the structural superfamily of organoheterocyclic compounds, synthesized by the sponge *Zyzzya fuliginosa.* Zyzzyanone B has previously been reported to possess cytotoxic activity and antioxidant effects [55,62]. 

**Figure 3:**
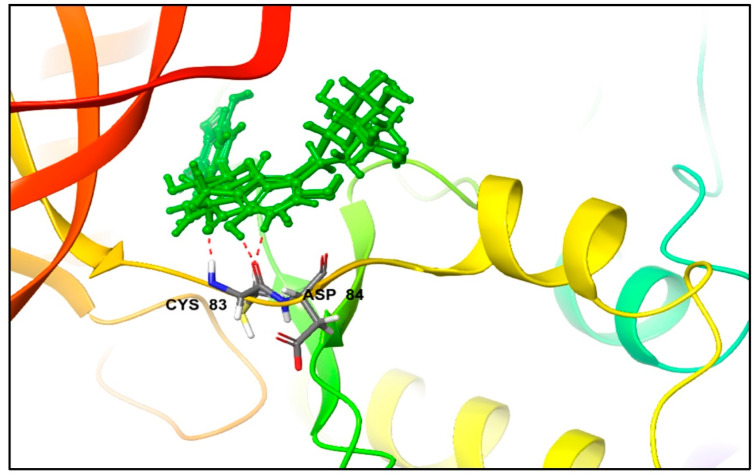
The 3D interactions of the three docked inhibitors (in green), overlayed as they are depicted, forming hydrogen bonds (red dashed lines) with Cys83 of the CDK5 enzyme (PDB ID: 7VDQ).

Furthermore, a closer examination of Table 2 reveals that both the co-crystallized reference inhibitor and excoecariphenol B established hydrophobic contacts with the same set of hydrophobic amino acid residues, namely Ile10, Val18, Ala31, Val64, Phe80, Phe82, Cys83, and Leu133. Additionally, excoecariphenol A reflected these contacts, except for Tyr15 and Val18, while zyzzyanone B maintained similar interactions, except for Val64. These observations highlight the potential binding similarities and distinctions among these compounds, shedding light on their interactions with the receptor. It has been previously documented that the active site of CDK5 closely resembles that of CDK1 and CDK2, posing a notable challenge in achieving selectivity. These three kinases share an almost identical ATP binding pocket. The only difference is that the residues Cys83 and Ser84 in CDK5 are Leu and Ser in CDK1, and Leu and His in CDK2. Importantly, there have been no reported instances of compounds interacting with these distinguishing features thus far. Of interest here, the residue Cys83 is engaged with our compounds (Figure 3) in hydrogen bonding, and this is a unique pattern of binding as there are no previously reported compounds acting similarly. 

Henceforth, we propose the possibility that the investigated compounds could potentially exhibit a distinctive selectivity for binding to CDK5. This selectivity is substantiated by their comparable binding energies and binding patterns when compared to the known co-crystallized inhibitor. This suggests the potential for these compounds to display a distinctive selectivity for CDK5 kinase activity. 

In order to strengthen their suitability in interacting with CDK5, subsequent molecular dynamics simulation is performed. 

### 3.2. ADME and Toxicity Analysis

Utilizing the QikProp tool within the Maestro software platform version 12.8, we conducted an assessment of the pharmacokinetic properties for the compounds that exhibited superior docking scores when compared to the co-crystallized inhibitor. This analytical step was undertaken with the primary objective of determining the druggability of the top-docked compounds and exploring their potential adherence to Lipinski’s rule of five criteria. As allocated by Lipinski’s rule, a molecule is deemed druggable when it complies with the following criteria: the molecular weight does not exceed 500 Da, has a maximum of 5 hydrogen bond donors (donorHB ≤ 5), has no more than 10 hydrogen bond acceptors (acceptorHB ≤ 10), and has a predicted octanol/water partition coefficient (QPlogPo/w) lower than 5 [63,64].

In Table 3, it can be noted that zyzzyanone B fulfills all the criteria that are set by Lipinski’s rule of five. On the other hand, excoecariphenol A and excoecariphenol B both show non-conformity to the rule by violating the prescribed limits of the number of hydrogen bond donors or acceptors. Moreover, the molecular weight of excoecariphenol B was 500.473 Da, which is slightly higher than the value set by the rule (500). Additionally, two parameters related to the potential for cellular membrane penetration were subject to scrutiny: QPlogBB, an indicative of blood–brain barrier permeability; and QPP Caco-2, reflecting cell membrane permeability. It is worth noting that all compounds under investigation demonstrated favorable cellular permeability characteristics while displaying restricted ability to traverse the blood–brain barrier.

Furthermore, a pivotal parameter, QPlog HERG, responsible for predicting IC50 values pertaining to the blockage of HERG K+ channels, as presented in Table 2, conveyed that none of the compounds exhibited indications of cardiotoxicity.

Within the framework of ProTox-II (Table 4), oral toxicity is characterized in terms of the median lethal dose (LD50), denoted as milligrams per kilogram of body weight for the test population. Notably, both excoecariphenol A and excoecariphenol B fall into class 5, exhibiting predicted LD50 values of 2500 mg/kg. Conversely, zyzzyanone B is categorized as class 4, possessing a calculated LD50 of 1000 mg/kg. These findings underscore the safety profile of these compounds when orally ingested. These toxicity findings are further potentiated by the fact that all three candidate compounds possess no hepatotoxicity, mutagenicity, cytotoxicity, carcinogenicity or immunogenicity.

### 3.3. Molecular Dynamics (MD) Simulations Analysis

Primary molecular docking provides initial intuitions into the nature of interaction between a small molecule and its target receptor, but it does not give an account to the flexibility of the protein–ligand complexes, i.e., it does not simulate the biological environment. Fortunately, the integration of molecular docking with highly comprehensive approaches like molecular dynamics simulation increases the simulation power and yields more realistic and comprehensive computational analysis [65]. MD simulation is introduced as a valuable tool for closely examining how macromolecular systems behave dynamically when exposed to small molecules. These simulations produce numerous trajectories, each offering a wealth of information about how proteins and ligands interact. These trajectories serve as a rich data source, enabling in-depth exploration and a deeper understanding of the intricate interactions within the protein–ligand system [66].

In this work, we maintained a molecular dynamics simulation for the top three compounds that were selectively filtered via docking scores and MM/GBSA free binding energy, specifically excoecariphenol B, excoecariphenol A, and zyzzyanone B. Additionally, the co-crystallized reference inhibitor was simulated and held as a control against which the other compounds were compared. The aforementioned simulations were conducted for 200 nanoseconds, since the CDK5 enzyme is a relatively large entity and is anticipated to fluctuate the most during a simulation run.

The analysis of MD is achieved using different metrics, for instance, the root means square deviation (RMSD), which is a fundamental technique employed in the examination of atomic positional variations within amino acid structures in the presence of a ligand. The RMSD essentially measures the average square root of the deviations from the mean distances observed within the protein–ligand complex. The inspection of molecular dynamics (MD) trajectories and the subsequent assessment of RMSD plots provide a valuable initial idea about the stability of simulated protein–ligand complexes, as previously indicated [67]. For a globular protein, maintaining stability in a simulated system typically entails fluctuations within a range of 1–3 Å. 

**Figure 4:**
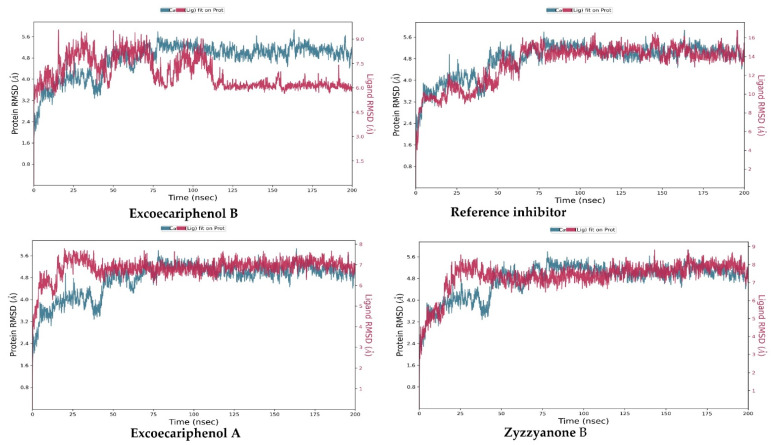
RMSD plotting of the top inhibitors and the co-crystallized reference inhibitor with the CDK5 enzyme (PDB ID: 7VDQ).

The molecular size greatly impacts the RMSD value; hence, as the size increases, it becomes difficult to directly address the MD simulation with a specific acceptable value. Importantly, as long as the RMSD value reaches a steady state of fluctuation, it can be fairly judged as a converged system [67]. For our studied compounds, Figure 4 depicts the RMSD plots for the four simulation runs during the 200 nanoseconds. It can be seen that all the compounds exhibited similar influence on the carbon alpha atoms of the protein, with an average value of 4.764 ± 0.64, indicating the occurrence of protein folding. Nonetheless, the general observation is that the protein fluctuated at the first 75 nanoseconds, then it converged and returned to fluctuate around the average value toward the simulation end-point. The fit of excoecariphenol B, excoecariphenol A, zyzzyanone B, and the co-crystallized reference inhibitor on the protein indicated that they converged after 125 ns, 40 ns, 25 ns and 75 ns, respectively.

The diversity in the behavior of amino acids and the precise sites of modifications within the protein are pivotal factors in comprehending the functional dynamics of the protein in the context of molecular dynamics simulations. Through a thorough examination of the trajectory data derived from root mean square fluctuation (RMSF) analysis, we can discern the dynamic behavior of individual amino acids [68]. 

**Figure 5:**
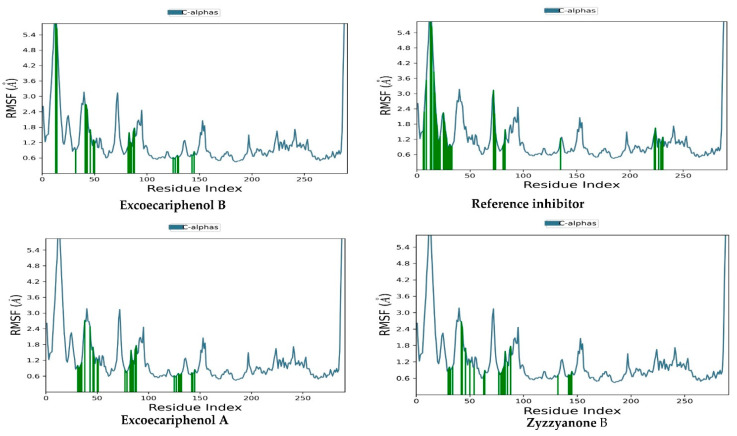
RMSF plotting of the top inhibitors and the co-crystallized reference inhibitor with the CDK5 enzyme (PDB ID: 7VDQ). Green lines represent loop regions.

The protein RMSF average values were 1.014 Å, 1.211 Å, 1.211 Å, and 1.211 Å for excoecariphenol B, excoecariphenol A, zyzzyanone B, and the co-crystallized reference inhibitor, respectively. As seen in Figure 5, there were areas of sharp peaks, particularly with excoecariphenol A as it presented contacts with Thr 14 and Tyr15, but providentially, they were brief and distant form the active site. The particular region of residues with a slight spike in the RMSF (up to 5 A) maybe due to the higher conformational flexibility of Thr14 and Tyr15. Fortunately, those residues are not essential for CDK5 binding. Overall, the RMFF patterns suggest that the simulated protein–ligand complexes are somewhat stable and with minimal fluctuations of the key amino acid residues.

**Figure 6:**
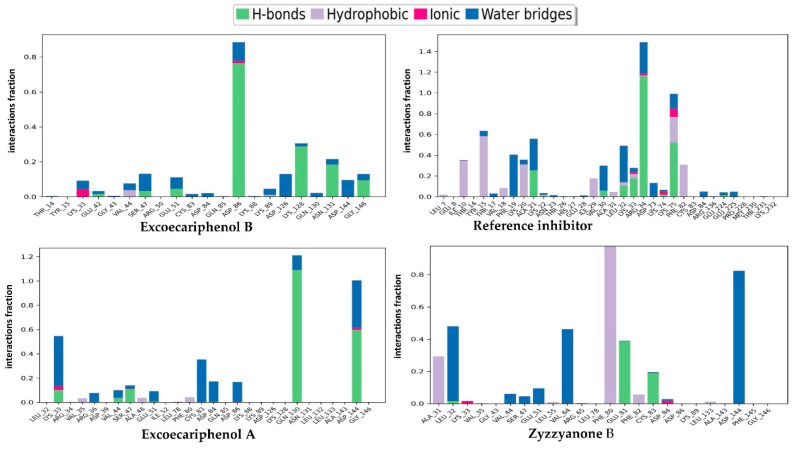
Protein–ligand contacts for the top three inhibitors and the co-crystallized reference inhibitor with the CDK5 enzyme (PDB ID: 7VDQ). The top legend depicts the color codes for the types of contacts that are formed throughout the simulation plotting of the top inhibitors and the enzyme.

The provided data in Figure 6 outline the various protein–ligand interactions observed during molecular dynamic simulations for three different compounds: excoecariphenol B, excoecariphenol A, and zyzzyanone B, as well as a reference inhibitor. 

For excoecariphenol B, hydrogen bonds are primarily formed with Asp86 (77%), indicating strong interactions, and to a lesser extent with Lys128 (30%), Asn131 (20%), and Gly146 (10%). Moreover, water bridges are observed with Lys33 (5%), Ser47 (8%), Glu51 (5%), Asp126 (10%), and Asp144 (8%), showing additional contributions to ligand–receptor interactions.

As for the reference inhibitor, direct hydrogen bonds are formed with several residues, including Arg34 (120%), indicating a strong interaction. Additionally, hydrogen bonds are established with Val30 (5%), Ala21 (22%), Leu32 (10%), Lys33 (15%), and Lys75 (45%). Water bridges, a type of interaction considered significant in ligand binding, are notable with Phe19 (40%), Ala21 (20%), Val30 (20%), Leu32 (30%), Arg34 (20%), and Lys75 (15%). Moreover, hydrophobic contacts, which are pivotal for stabilizing ligand–receptor complexes, are present with Ile10 (35%), Tyr15 (60%), Kys20 (30%), Ile29 (15%), Lys75 (20%), and Phe82 (20%). An ionic interaction with Lys75 (5%) further contributes to the overall binding.

The compound excoecariphenol A is engaged in direct hydrogen bonds with Lys33 (10%), Ser47 (10%), Gln130 (110%), and Asp144 (60%), reflecting substantial interactions. In addition, water bridges are noted with Lys33 (50%), Arg36 (5%), Val44 (5%), Glu51 (8%), Cys83 (35%), Asp84 (20%), Asp86 (20%), Gln130 (5%), and Asp144 (40%), indicating their involvement in the ligand binding process. Notably, Gln130 is involved in both direct hydrogen bonds and water bridges.

For zyzzyanone B, direct hydrogen bonds are significantly established with Glu81 (140%) and Cys83 (20%), underlining strong binding. Alongside that, water bridges are prominent with Leu32 (45%), Val44 (5%), Ser47 (5%), Glu51 (7%), Val64 (45%), and Asp144 (80%). Moreover, hydrophobic contacts are present with Phe80 (100%), Phe82 (5%), and Ala31 (25%), highlighting their role in stabilization.

In comparison, excoecariphenol B forms strong hydrogen bonds with Asp86, while the reference inhibitor demonstrates a robust interaction with Arg34. Excoecariphenol A and zyzzyanone B also establish noteworthy hydrogen bonds primarily with Gln130 and Glu81, respectively. In addition, all compounds engage in water bridge interactions. Excoecariphenol A and zyzzyanone B exhibit prominent water bridge interactions with various residues. In contrast, excoecariphenol B and the reference inhibitor demonstrate water bridges with fewer residues. Conversely, hydrophobic interactions are not evident for all compounds. The reference inhibitor displayed significant hydrophobic contacts, especially with Tyr15 and Phe80. Zyzzyanone B forms a substantial hydrophobic interaction with Phe80. 

In summary, each compound exhibits unique interaction patterns with the receptor, suggesting varying modes of binding. Excoecariphenol B and the reference inhibitor prioritize hydrogen bonding, while excoecariphenol A and zyzzyanone B rely on a combination of hydrogen bonding, water bridges, and hydrophobic contacts. These distinctive interaction profiles may have implications for the compounds’ selectivity and potency as ligands, emphasizing the importance of understanding these interactions in drug design and development.

**Figure 7:**
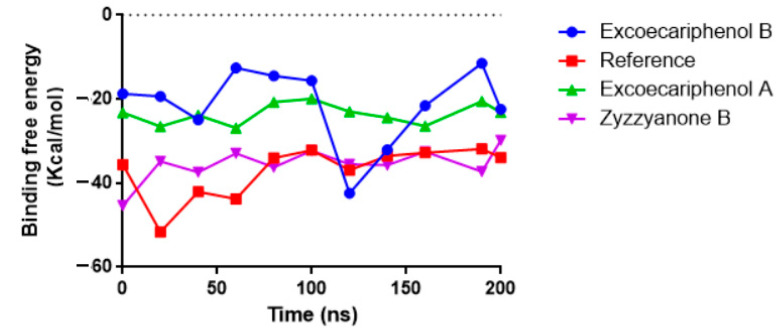
Post-MD MM/GBSA curves for the co-crystallized reference inhibitor and the top-three simulated compounds with the CDK5 target.

Furthermore, in order to measure the total energy after MD production, post-MD MM/GBSA calculations were performed. The co-crystallized reference and the best three compounds’ trajectories underwent MM/GBSA calculations. Due to the substantial computational expense, only 10 frames were chosen from a total of 2000 frames. As can be noted in Figure 7, the reference showed the lowest energy value, with a mean value of −37.12 ± 6.212 kcal/mol. On the other hand, excoecariphenol B, excoecariphenol A, and zyzzyanone B presented energy averages of −21.42 ± 9.155 kcal/mol, −23.51 ± 2.453 kcal/mol, and −35.45 ± 4.042 kcal/mol, respectively. Notably, zyzzyanone B is the lowest of the candidate compounds, implying its superior binding.

## 4. Conclusions

CDK inhibitors hold immense promise in halting tumor development, progression, and metastasis, mediated through diverse pathways such as CDK5–FAK, PPARγ, PP2A, Hippo, and Wnt/β-catenin, among others. Despite growing demand, the clinical landscape has yet to witness the emergence of a selective CDK5 inhibitor. This research sought to address this gap by employing a range of computational techniques. Our study involved the screening of a substantial library of 47,450 marine natural products employing a multi-stage Glide docking process (HTVS, SP, and XP). Subsequently, the top 10 docked complexes were subjected to careful refinement via MM/GBSA analysis. Among these, three candidate compounds, specifically excoecariphenol B, excoecariphenol A, and zyzzyanone B, exhibited promising attributes. Through the extensive 200 nanoseconds of molecular dynamics simulations, we scrutinized the stability of these compounds, further enhancing our confidence in their potential as CDK5 inhibitors. Taken together, our in silico methodologies suggest that these marine-based compounds require further experimental investigation to ascertain their selectivity and therapeutic efficacy as novel CDK5 inhibitors. This research endeavors to contribute to the ongoing quest for innovative anti-cancer therapies, with a particular focus on CDK5 inhibition.

## Figures and Tables

**Table 2:** Summary of the types of interactions that are formed by the docked inhibitors.

Compound	Hydrogen Bonds		Hydrophobic Interactions	Other Interactions
	Residue	Distance (Å)		
**Excoecariphenol B**	Gln51 Cys83 Asp144	1.93 1.70 2.39	Ile10, Tyr15, Val18, Ala31, Val64, Phe80, Phe82, Cys83, and Leu133	-
**Excoecariphenol A**	Gln51 Cys83	1.83 1.68	Ile10, Ala31, Val64, Phe80, Phe82, Cys83, and Leu133	-
**Zyzzyanone B**	Gln51 Glu81 Asp86 Cys83	1.85 2.25 2.11 1.94, 1.67	Ile10, Tyr15, Val18, Ala31, Phe80, Phe82, Cys83, and Leu133	salt bridge-Asp86 Pi-Pi stacking-Phe80
**Reference**	Lys33 Gln51 Cys83 Asp86 Asp144	2.76 1.87 1.97, 1.84 2.08 1.98, 2.39	Ile10, Tyr15, Val18, Ala31, Val64, Phe80, Phe82, Cys83, and Leu133	salt bridge-Asp144 Pi-cation-Tyr15

**Table 3:** Predicted ADME parameters for the top three compounds.

	HBD ^a^	HBA ^b^	QPlog Po/w ^c^	QPlog S ^d^	QPlog HERG ^e^	QPP Caco ^f^	QPlog BB ^g^	Mwt ^h^	Rule Of Five ^i^
**Excoecariphenol B**	10	15.2	−2.033	−2.489	−5.025	1.377	−4.213	500.473	3
**Excoecariphenol A**	9	14.45	−1.406	−2.741	−5.305	4.115	−3.69	484.474	2
**Zyzzyanone B**	4	6.25	1.076	−3.043	−6.275	22.892	−1.397	335.362	0
**Standard values**	≤5	≤10	−2.0–6.5	−6.5˗0.5	Below −5	>25 poor <500 great	−3–1.2	>500	0˗4

Note: (a) HBD (hydrogen bond donor). (b) HBA (hydrogen bond acceptor). (c) Predicted LogP (partition coefficient in octanol/water). (d) Predicted aqueous solubility. (e) Predicted IC50 for HERG K+ blockade. (f) Predicted Caco-2 cell permeability. (g) Predicted blood–brain partition coefficient. (h) Molecular weight. (i) Number of Lipinski’s rule of five violations.

**Table 4:** Predicted toxicity parameters for the top three compounds.

Compound	Oral Toxicity		Organ Toxicity	Toxicity Endpoints Prediction			
	Toxicity Class	Predicted LD50 (mg/kg)	Hepatotoxicity	Mutagenicity	Cyto Toxicity	Carcinogenicity	Immunotoxicity
**Excoecariphenol B**	5	2500	inactive	inactive	inactive	inactive	inactive
**Excoecariphenol A**	5	2500	inactive	inactive	inactive	inactive	inactive
**Zyzzyanone B**	4	1000	inactive	inactive	inactive	inactive	inactive

## Data Availability

The raw data presented in this study are available on public databases mentioned in the article, and the derived data are available on request from the corresponding author. The data are not publicly available due to confidentiality agreement with research.

## Abbreviations

ATP	Adenosine triphosphate
CDK5	Cyclin-dependent kinase 5
CHD	Coronary heart disease
CMNPD	Comprehensive marine natural product database
FDA	Food and Drug Administration
HTVS	High-throughput virtual screening
MD	Molecular dynamics
MM/GBSA	Molecular mechanics/generalized born surface area
NPT	Isothermal–isobaric
OPLS	Optimized potentials for liquid simulations
RMSD	Root means square deviation
RMSF	Root mean square fluctuation
TIP3P	Transferable interaction potential
PDB	Protein data bank
SP	Standard precision
XP	Extra precision
